# Crystal structure of 3-{5-[3-(4-fluoro­phen­yl)-1-isopropyl-1*H*-indol-2-yl]-1*H*-pyrazol-1-yl}indolin-2-one ethanol monosolvate

**DOI:** 10.1107/S2056989016001614

**Published:** 2016-02-03

**Authors:** Md. Lutfor Rahman, Ajaykumar D. Kulkarni, Mashitah Mohd. Yusoff, Huey Chong Kwong, Ching Kheng Quah

**Affiliations:** a University Malaysia Pahang, Faculty of Industrial Sciences and Technology, 26300 Gambang, Kuantan, Pahang, Malaysia; b Department of Chemistry, KLS’s Gogte Institute of Technology, Jnana Ganga, Udyambag, Belagavi-590008 Karnataka, India; c School of Chemical Sciences, Universiti Sains Malaysia, 11800 USM, Penang, Malaysia; d X-ray Crystallography Unit, School of Physics, Universiti Sains Malaysia, 11800 USM, Penang, Malaysia

**Keywords:** crystal structure, indol-2-one, pyrazole, indole, Schiff base, N—H⋯O and O—H⋯O hydrogen bonds, C—H⋯π inter­actions

## Abstract

The title indolin-2-one compound, C_28_H_23_FN_4_O·C_2_H_6_O, crystallizes as a 1:1 ethanol solvate. The ethanol mol­ecule is disordered over two positions with refined site occupancies of 0.560 (14) and 0.440 (14). The pyrazole ring makes dihedral angles of 84.16 (10) and 85.33 (9)° with the indolin-2-one and indole rings, respectively, whereas the dihedral angle between indolin-2-one and indole rings is 57.30 (7)°. In the crystal, the components are linked by N—H⋯O and O—H⋯O hydrogen bonds, forming an inversion mol­ecule–solvate 2:2 dimer with *R*_4_^4^(12) ring motifs. The crystal structure is consolidated by π–π inter­action between pairs of inversion-related indolin-2-one rings [inter­planar spacing = 3.599 (2) Å].

## Abstract 2

The title compound crystallizes as a 1:1 ethanol solvate, with the pyrazole ring almost normal to both of the indol-2-one ring and indole rings. In the crystal, mol­ecules are linked by pairs of N—H⋯O and O—H⋯O hydrogen bonds, forming an inversion mol­ecule–solvate dimer with an 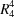
(12) ring motif.

## Chemical context   

Heterocyclic compounds containing the pyrazolone nucleus, indole, and its derivatives play an important role in biological activities. The synthesis and biological activity of some new indole derivatives containing a pyrazole moiety have been reported (Raju *et al.*, 2013 ▸). Pyrazole and its analogues have been found to exhibit industrial and biologically active applications (el-Kashef *et al.*, 2000 ▸; Taha *et al.*, 2001 ▸; Brzozowski & Sączewski,, 2002 ▸). Consequently, synthesis of indole derivatives has been a major topic in organic and medicinal chemistry over the past few decades. Nitro­gen-containing heterocycles are universal systems in nature and are consequently considered as privileged structures in drug discovery (Raju *et al.*, 2013 ▸). A literature survey shows that some pyrazoles plays an essential role in biologically active compounds and also in medicinal chemistry (Penning *et al.*, 2006 ▸), exhibiting phenomena such as anti­bacterial (Pevarello *et al.*, 2006 ▸), anti­fungal, anti­viral (Meghashyam *et al.*, 2011 ▸), anti-oxidant (Singarave & Sarkkarai, 2011 ▸), anti-inflammatory (Mana *et al.*, 2010 ▸), and anti­cancer (Pathak *et al.*, 2010 ▸) effects *etc*. Certain indole derivatives have also been reported to exhibit wide-spectrum activities such as anti­parkinsonian and anti­convulsant effects (Siddiqui *et al.*, 2008 ▸; Archana *et al.*, 2002 ▸). In addition, pyrazoles have played a crucial role in the development of theory in heterocyclic chemistry, and are also used extensively as useful synthons in organic synthesis. Isatin, an endogenous indole and its derivatives have been shown to exhibit a wide range of biological activities (Daisley & Shah, 1984 ▸; Pandeya *et al.*, 1999 ▸). In addition, the biological significance of fluvastatin, an indole derivative, is well established (Repič *et al.*, 2001 ▸). As part of our studies in this area, we now present a pyrazole as a central unit linked with 3-[3-(4-fluoro­phen­yl)-1-iso­propyl­indolin-2-yl]acryl­aldehyde and 3-hydrazonoindolin-2-one, synthesized according to a procedure reported in the literature (Elkanzi, 2013 ▸).
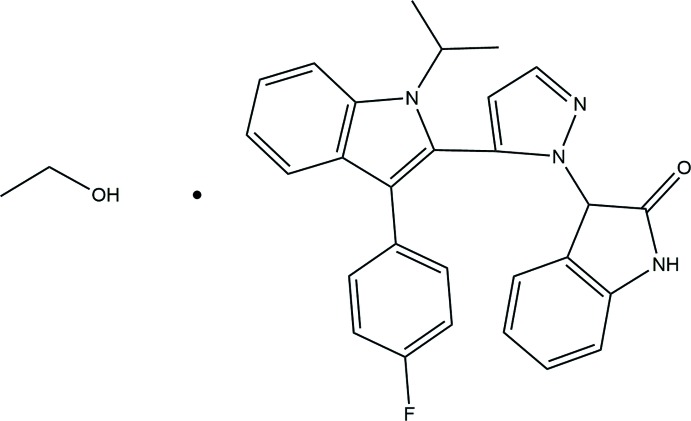


## Structural commentary   

**Figure 1:**
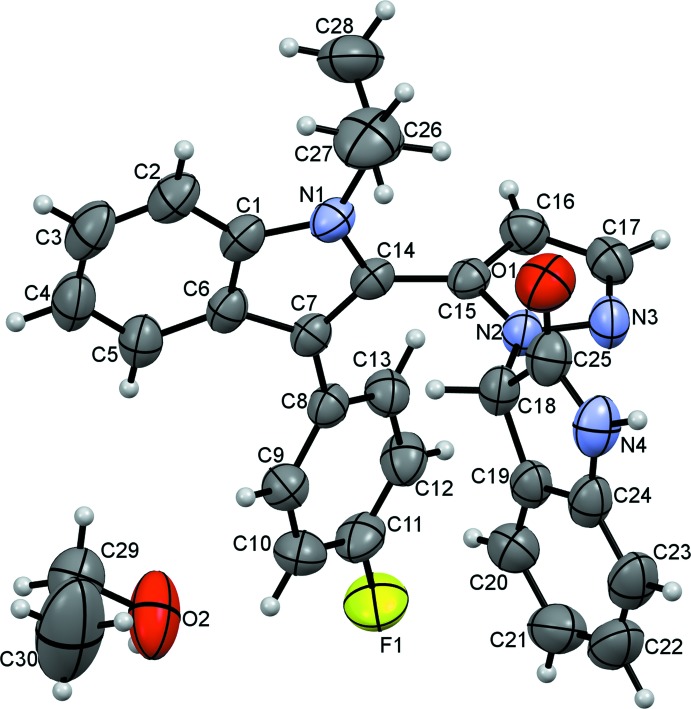
The mol­ecular structure of the title compound. Displacement ellipsoids are drawn at the 30% probability level. Only the major component of the disordered ethanol solvent mol­ecule is shown.

The asymmetric unit of the title compound (Fig. 1 ▸) comprises of a 3-{5-[3-(4-fluoro­phen­yl)-1-isopropyl-1*H*-indol-2-yl]-1*H*-pyrazol-1-yl}indolin-2-one and an ethanol solvent mol­ecule. The pyrrolidin-2-one ring has an essentially planar conformation, with maximum deviation from the mean plane of the ring of 0.04 (2) Å at C25. The pyrazole ring is almost planar [maximum deviation of ±0.006 (2) Å for atoms N2 and C15], as are the fluoro­phenyl [maximum deviation of ± 0.011 (2) Å for atoms C10 and C13] and indole [maximum deviation of ± 0.0019 (2) Å for atom C14] rings. The connecting pyrazole ring is almost normal to both indol-2-one and indole rings with dihedral angles of 84.16 (10)° and 85.33 (9)°, respectively, while the indole and fluoro­phenyl rings are tilted toward one another by 40.74 (8)°. The bond lengths and angles in the fluoro­phenyl-indole moiety of the title mol­ecule are comparable to those of previously reported compounds (Kulkarni *et al.*, 2015*a* ▸,*b* ▸).

## Supra­molecular features   

**Figure 2:**
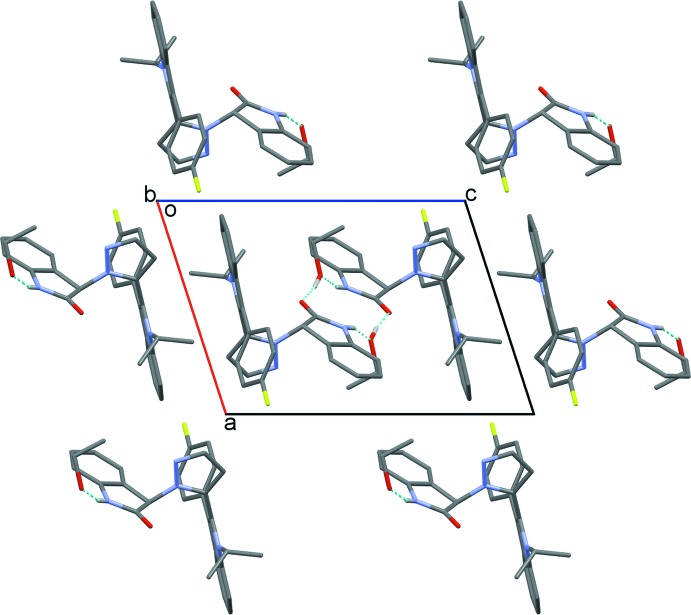
The crystal packing of the title compound viewed along the *b* axis. The N—H⋯O and O—H⋯O hydrogen bonds are shown as dashed lines. H atoms not involved in hydrogen bonding have been omitted for clarity.

In the crystal, the main mol­ecules and ethanol solvate mol­ecules are linked *via* pairs of N4—H1*N*1⋯O2 and O2—H1*O*2⋯O1 hydrogen bonds (Table 1 ▸), forming an inversion-related mol­ecule-solvate 2:2 dimer with an 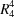
(12) ring motif (Fig. 2 ▸) (Bernstein *et al.*, 1995 ▸). The crystal structure also features π–π inter­actions between pairs of inversion-related (1 − *x*, 1 − *y*, 1 − *z*) indolin-2-one rings with an inter­planar spacing of 3.599 (2) Å.

## Database survey   

A search of the Cambridge Structural Database (CSD, Version 35.6, last update May 2015; Groom & Allen, 2014 ▸) using 4-(λ^1^-azan­yl)-5-methyl-2,4-di­hydro-3*H*-1,2,4-triazole-3-thione as the main skeleton, revealed the presence of 57 structures containing the triazole-thione moiety but only four structures containing the fluvastatin nucleus. These include 5-[3-(4-fluoro­phen­yl)-1-isopropyl-1*H*-indol-2-yl]-1-(*X*)penta-2,4-diene-1-one, where *X* = 4-nitro­phenyl (NUHNAH), 2-hy­droxy­phenyl (NUHNEL), 4-meth­oxy­phenyl (NUHNIP) and 4-chloro­phenyl (NUHNOV) (Kalalbandi *et al.*, 2015 ▸). In these four compounds, the 4-fluoro­phenyl ring of the fluvastatin nucleus is inclined to the indole ring by dihedral angles ranging from *ca* 46.66 to 68.59°, compared to 40.74 (8)° for the title compound.

## Synthesis and crystallization   

The title compound was synthesized by refluxing a hot methano­lic solution (30 mL) of 3-(3-(4-fluoro­phen­yl)-1-iso­propyl­indolin-2-yl)acryl­aldehyde (0.01mol) and a hot methano­lic solution (30 mL) of 3-hydrazonoeindolin-2-one (0.01mol) for 5 h with addition of 4 drops of conc. hydro­chloric acid (Ajaykumar *et al.*, 2009 ▸). The product obtained after evaporation of the solvent was filtered, washed with cold MeOH and recrystallized from ETOH. The single crystal used for the crystal analysis was grown by the slow evaporation of a solution in chloro­form–ethanol (1:1). Yield (m.p.): 78% (551 K). ^1^HNMR (CDCl_3_) in p.p.m.: 7.94 (*s*, 1H, NH, indole), 7.76 (*d*, 1H, Ar-H), 7.72 (*m*, 2H, Ar–H), 7.37 (*m*, 2H, Ar-H), 7.32 (*t*, 1H, Ar-H), 7.20 (*t*, 1H, Ar-H), 7.13 (*d*, 1H, Ar-H), 7.10 (*d*, 2H, Ar-H), 6.77 (*t*, 1H, Ar-H), 6.70 (*d*, 1H, Ar-H), 6.67 (*d*, 1H, pyrazole), 5.48 (*d*, 2H, pyrazole), 5.37 (*s*, 1H, indole), 4.73 (*m*, 1H, isoprop­yl), 1.73 (*m*, 6H, meth­yl). IR (KBr) cm^−1^: 3250 (N—H, indole), 2827 (–CH_3_), 1720 (C=O, ketone), 1618 (C=C, Ar), 1520 (C—C, Ar), 1469 (–CH_3_), 1221 (C—N).

## Refinement   

Crystal data, data collection and structure refinement details are summarized in Table 2 ▸. The ethanol mol­ecule is disordered over two positions with refined site occupancies of 0.560 (14): 0.440 (14). The disorder components were restrained to have similar geometry. The N-bound H atom was located in a difference Fourier map and freely refined. The C-bound H atoms were positioned geometrically (C—H = 0.93–0.98 Å) and refined using a riding model with *U*_iso_(H) = 1.5*U*_eq_(C-meth­yl) and 1.2*U*_eq_(C) for other H atoms.

## Supplementary Material

Crystal structure: contains datablock(s) global, I. DOI: 10.1107/S2056989016001614/pk2572sup1.cif

Structure factors: contains datablock(s) I. DOI: 10.1107/S2056989016001614/pk2572Isup2.hkl

Supporting information file. DOI: 10.1107/S2056989016001614/pk2572Isup3.cml

CCDC reference: 1450044

Additional supporting information:  crystallographic information; 3D view; checkCIF report

## Figures and Tables

**Table 1:** Hydrogen-bond geometry (Å, °)

*D*—H⋯*A*	*D*—H	H⋯*A*	*D*⋯*A*	*D*—H⋯*A*
N4—H1*N*1⋯O2^i^	0.85 (2)	1.92 (3)	2.750 (19)	165 (2)
O2—H1*O*2⋯O1^ii^	0.98 (9)	1.67 (9)	2.650 (2)	172 (11)

Symmetry codes: (i) 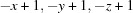; (ii) 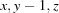.

**Table 2:** Experimental details

Crystal data	
Chemical formula	C_28_H_23_FN_4_O·C_2_H_6_O
*M*_r_	496.57
Crystal system, space group	Triclinic, *P* 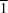
Temperature (K)	297
*a*, *b*, *c* (Å)	9.9754 (8), 10.2139 (8), 14.0294 (11)
α, β, γ (°)	75.7386 (15), 71.0062 (14), 83.1264 (14)
*V* (Å^3^)	1308.73 (18)
*Z*	2
Radiation type	Mo *K*α
μ (mm^−1^)	0.09
Crystal size (mm)	0.42 × 0.22 × 0.22

Data collection	
Diffractometer	Bruker APEXII DUO CCD area detector
Absorption correction	Multi-scan (*SADABS*; Bruker, 2009 ▸)
*T*_min_, *T*_max_	0.884, 0.955
No. of measured, independent and observed [*I* > 2σ(*I*)] reflections	32072, 5778, 3733
*R*_int_	0.032
(sin θ/λ)_max_ (Å^−1^)	0.650

Refinement	
*R*[*F* ^2^ > 2σ(*F* ^2^)], *wR*(*F* ^2^), *S*	0.057, 0.130, 1.21
No. of reflections	5778
No. of parameters	375
No. of restraints	3
H-atom treatment	H atoms treated by a mixture of independent and constrained refinement
Δρ_max_, Δρ_min_ (e Å^−3^)	0.15, −0.19

Computer programs: *APEX2* (Bruker, 2009 ▸), *SAINT* (Bruker, 2009 ▸), *SHELXS97* (Sheldrick, 2008 ▸), *SHELXL2013* (Sheldrick, 2015 ▸), *Mercury* (Macrae *et al.*, 2008 ▸) and *PLATON* (Spek, 2009 ▸).

## supplementary crystallographic information

### Crystal data

**Table:**

C_28_H_23_FN_4_O·C_2_H_6_O	*Z* = 2
*M**_r_* = 496.57	*F*(000) = 524
Triclinic, *P*1	*D*_x_ = 1.260 Mg m^−^^3^
*a* = 9.9754 (8) Å	Mo *K*α radiation, λ = 0.71073 Å
*b* = 10.2139 (8) Å	Cell parameters from 9792 reflections
*c* = 14.0294 (11) Å	θ = 2.3–27.6°
α = 75.7386 (15)°	µ = 0.09 mm^−^^1^
β = 71.0062 (14)°	*T* = 297 K
γ = 83.1264 (14)°	Block, colourless
*V* = 1308.73 (18) Å^3^	0.42 × 0.22 × 0.22 mm

### Data collection

**Table:**

Bruker APEXII DUO CCD area-detector diffractometer	5778 independent reflections
Radiation source: fine-focus sealed tube	3733 reflections with *I* > 2σ(*I*)
Graphite monochromator	*R*_int_ = 0.032
φ and ω scans	θ_max_ = 27.5°, θ_min_ = 1.6°
Absorption correction: multi-scan (*SADABS*; Bruker, 2009)	*h* = −12→12
*T*_min_ = 0.884, *T*_max_ = 0.955	*k* = −13→13
32072 measured reflections	*l* = −18→18

### Refinement

**Table:**

Refinement on *F*^2^	3 restraints
Least-squares matrix: full	Hydrogen site location: mixed
*R*[*F*^2^ > 2σ(*F*^2^)] = 0.057	H atoms treated by a mixture of independent and constrained refinement
*wR*(*F*^2^) = 0.130	*w* = 1/[σ^2^(*F*_o_^2^) + (0.0361*P*)^2^ + 0.3328*P*] where *P* = (*F*_o_^2^ + 2*F*_c_^2^)/3
*S* = 1.21	(Δ/σ)_max_ < 0.001
5778 reflections	Δρ_max_ = 0.15 e Å^−^^3^
375 parameters	Δρ_min_ = −0.19 e Å^−^^3^

### Special details

**Table:**

Geometry. All esds (except the esd in the dihedral angle between two l.s. planes) are estimated using the full covariance matrix. The cell esds are taken into account individually in the estimation of esds in distances, angles and torsion angles; correlations between esds in cell parameters are only used when they are defined by crystal symmetry. An approximate (isotropic) treatment of cell esds is used for estimating esds involving l.s. planes.

### Fractional atomic coordinates and isotropic or equivalent isotropic displacement parameters (Å^2^)

**Table:**

	*x*	*y*	*z*	*U*_iso_*/*U*_eq_	Occ. (<1)
F1	0.95103 (15)	0.08376 (14)	0.14670 (13)	0.0965 (5)	
N1	0.37863 (16)	0.67252 (16)	0.16316 (12)	0.0496 (4)	
H1N1	0.596 (2)	0.795 (2)	0.5053 (16)	0.059*	
N2	0.66480 (15)	0.68580 (15)	0.23475 (11)	0.0477 (4)	
N3	0.77904 (17)	0.76065 (17)	0.21225 (13)	0.0571 (4)	
N4	0.5929 (2)	0.7342 (2)	0.47443 (14)	0.0662 (5)	
O1	0.47523 (17)	0.85895 (18)	0.36321 (13)	0.0801 (5)	
C1	0.28456 (19)	0.5768 (2)	0.17372 (14)	0.0500 (5)	
C2	0.1405 (2)	0.5909 (3)	0.18238 (16)	0.0638 (6)	
H2A	0.0924	0.6749	0.1806	0.077*	
C3	0.0725 (2)	0.4771 (3)	0.19350 (18)	0.0732 (7)	
H3A	−0.0236	0.4841	0.1996	0.088*	
C4	0.1431 (2)	0.3511 (3)	0.19594 (18)	0.0706 (6)	
H4A	0.0935	0.2756	0.2038	0.085*	
C5	0.2849 (2)	0.3364 (2)	0.18689 (16)	0.0587 (5)	
H5A	0.3316	0.2518	0.1882	0.070*	
C6	0.35815 (19)	0.45041 (19)	0.17569 (13)	0.0468 (4)	
C7	0.50280 (18)	0.47104 (18)	0.16459 (13)	0.0436 (4)	
C8	0.62068 (19)	0.36889 (18)	0.16080 (14)	0.0452 (4)	
C9	0.6011 (2)	0.2407 (2)	0.22518 (16)	0.0565 (5)	
H9A	0.5122	0.2197	0.2725	0.068*	
C10	0.7109 (2)	0.1441 (2)	0.22023 (19)	0.0671 (6)	
H10A	0.6964	0.0579	0.2624	0.081*	
C11	0.8413 (2)	0.1780 (2)	0.15208 (19)	0.0640 (6)	
C12	0.8657 (2)	0.3021 (2)	0.08737 (18)	0.0613 (5)	
H12A	0.9556	0.3225	0.0416	0.074*	
C13	0.75482 (19)	0.3966 (2)	0.09108 (16)	0.0522 (5)	
H13A	0.7699	0.4809	0.0460	0.063*	
C14	0.51043 (18)	0.60667 (18)	0.15671 (13)	0.0432 (4)	
C15	0.63180 (18)	0.68060 (17)	0.14957 (14)	0.0428 (4)	
C16	0.7318 (2)	0.75366 (19)	0.06796 (15)	0.0547 (5)	
H16A	0.7398	0.7686	−0.0018	0.066*	
C17	0.8187 (2)	0.8008 (2)	0.11080 (16)	0.0549 (5)	
H17A	0.8957	0.8543	0.0725	0.066*	
C18	0.5866 (2)	0.6370 (2)	0.34278 (14)	0.0502 (5)	
H18A	0.5028	0.5904	0.3490	0.060*	
C19	0.6725 (2)	0.5487 (2)	0.40595 (14)	0.0523 (5)	
C20	0.7444 (2)	0.4273 (2)	0.39863 (18)	0.0666 (6)	
H20A	0.7423	0.3823	0.3490	0.080*	
C21	0.8208 (3)	0.3727 (3)	0.4674 (2)	0.0833 (7)	
H21A	0.8715	0.2906	0.4632	0.100*	
C22	0.8225 (3)	0.4381 (3)	0.5413 (2)	0.0877 (8)	
H22A	0.8746	0.3998	0.5863	0.105*	
C23	0.7486 (3)	0.5592 (3)	0.55029 (17)	0.0770 (7)	
H23A	0.7489	0.6030	0.6010	0.092*	
C24	0.6745 (2)	0.6130 (2)	0.48173 (15)	0.0583 (5)	
C25	0.5419 (2)	0.7586 (2)	0.39376 (17)	0.0593 (5)	
C26	0.3559 (2)	0.8204 (2)	0.14204 (17)	0.0607 (5)	
H26A	0.4430	0.8576	0.1401	0.073*	
C27	0.2370 (3)	0.8682 (3)	0.2271 (2)	0.0910 (8)	
H27A	0.2350	0.9651	0.2145	0.137*	
H27B	0.2526	0.8299	0.2925	0.137*	
H27C	0.1480	0.8401	0.2281	0.137*	
C28	0.3389 (3)	0.8721 (3)	0.0352 (2)	0.0960 (9)	
H28A	0.3347	0.9691	0.0190	0.144*	
H28B	0.2529	0.8400	0.0341	0.144*	
H28C	0.4183	0.8397	−0.0149	0.144*	
O2	0.3483 (14)	0.060 (2)	0.4525 (12)	0.115 (5)	0.560 (14)
H1O2	0.401 (10)	−0.016 (8)	0.423 (8)	0.138*	0.560 (14)
C29	0.1962 (7)	0.0616 (11)	0.4632 (7)	0.088 (2)	0.560 (14)
H29A	0.1654	0.1480	0.4274	0.106*	0.560 (14)
H29B	0.1769	−0.0096	0.4353	0.106*	0.560 (14)
C30	0.1250 (16)	0.039 (2)	0.5740 (8)	0.186 (9)	0.560 (14)
H30A	0.0243	0.0400	0.5871	0.279*	0.560 (14)
H30B	0.1468	0.1100	0.6001	0.279*	0.560 (14)
H30C	0.1569	−0.0464	0.6078	0.279*	0.560 (14)
O2A	0.3340 (15)	0.080 (2)	0.4359 (12)	0.077 (3)	0.440 (14)
H2O2	0.384 (13)	0.024 (9)	0.415 (9)	0.092*	0.440 (14)
C29A	0.2173 (11)	−0.0028 (13)	0.5152 (12)	0.101 (4)	0.440 (14)
H29C	0.1718	−0.0491	0.4822	0.121*	0.440 (14)
H29D	0.2550	−0.0699	0.5632	0.121*	0.440 (14)
C30A	0.1171 (11)	0.089 (2)	0.5688 (13)	0.128 (6)	0.440 (14)
H30D	0.0319	0.0425	0.6102	0.192*	0.440 (14)
H30E	0.0953	0.1644	0.5194	0.192*	0.440 (14)
H30F	0.1573	0.1195	0.6124	0.192*	0.440 (14)

### Atomic displacement parameters (Å^2^)

**Table:**

	*U*^11^	*U*^22^	*U*^33^	*U*^12^	*U*^13^	*U*^23^
F1	0.0752 (9)	0.0731 (9)	0.1446 (14)	0.0267 (7)	−0.0456 (9)	−0.0283 (9)
N1	0.0461 (9)	0.0524 (9)	0.0552 (9)	0.0063 (8)	−0.0226 (7)	−0.0154 (7)
N2	0.0442 (9)	0.0535 (9)	0.0478 (9)	−0.0089 (7)	−0.0156 (7)	−0.0103 (7)
N3	0.0507 (10)	0.0629 (11)	0.0610 (11)	−0.0173 (8)	−0.0171 (8)	−0.0129 (8)
N4	0.0666 (12)	0.0836 (14)	0.0578 (11)	−0.0107 (10)	−0.0164 (9)	−0.0328 (10)
O1	0.0743 (11)	0.0801 (11)	0.0922 (12)	0.0141 (9)	−0.0286 (9)	−0.0347 (10)
C1	0.0439 (11)	0.0653 (12)	0.0441 (10)	0.0005 (9)	−0.0173 (8)	−0.0139 (9)
C2	0.0469 (12)	0.0835 (16)	0.0632 (13)	0.0037 (11)	−0.0217 (10)	−0.0167 (11)
C3	0.0428 (12)	0.110 (2)	0.0685 (15)	−0.0097 (13)	−0.0182 (11)	−0.0177 (14)
C4	0.0574 (14)	0.0905 (18)	0.0691 (15)	−0.0241 (13)	−0.0209 (11)	−0.0151 (12)
C5	0.0551 (12)	0.0682 (13)	0.0579 (12)	−0.0115 (10)	−0.0187 (10)	−0.0172 (10)
C6	0.0435 (10)	0.0590 (12)	0.0415 (10)	−0.0048 (9)	−0.0152 (8)	−0.0136 (8)
C7	0.0439 (10)	0.0496 (10)	0.0415 (10)	−0.0010 (8)	−0.0165 (8)	−0.0139 (8)
C8	0.0456 (10)	0.0481 (11)	0.0489 (11)	−0.0003 (8)	−0.0200 (8)	−0.0168 (9)
C9	0.0552 (12)	0.0539 (12)	0.0599 (12)	−0.0033 (10)	−0.0186 (10)	−0.0103 (10)
C10	0.0733 (15)	0.0502 (12)	0.0804 (16)	0.0023 (11)	−0.0345 (13)	−0.0066 (11)
C11	0.0553 (13)	0.0572 (13)	0.0909 (17)	0.0167 (11)	−0.0365 (12)	−0.0273 (12)
C12	0.0465 (12)	0.0613 (13)	0.0792 (15)	0.0015 (10)	−0.0182 (10)	−0.0244 (12)
C13	0.0480 (11)	0.0476 (11)	0.0623 (12)	−0.0003 (9)	−0.0169 (9)	−0.0156 (9)
C14	0.0428 (10)	0.0486 (10)	0.0417 (10)	0.0023 (8)	−0.0172 (8)	−0.0126 (8)
C15	0.0440 (10)	0.0412 (10)	0.0469 (10)	0.0044 (8)	−0.0189 (8)	−0.0131 (8)
C16	0.0605 (12)	0.0545 (12)	0.0474 (11)	−0.0048 (10)	−0.0153 (10)	−0.0085 (9)
C17	0.0509 (11)	0.0497 (11)	0.0587 (13)	−0.0071 (9)	−0.0102 (10)	−0.0091 (9)
C18	0.0468 (11)	0.0604 (12)	0.0463 (11)	−0.0129 (9)	−0.0132 (9)	−0.0133 (9)
C19	0.0498 (11)	0.0611 (12)	0.0459 (11)	−0.0156 (10)	−0.0143 (9)	−0.0056 (9)
C20	0.0711 (14)	0.0623 (14)	0.0647 (14)	−0.0098 (12)	−0.0221 (12)	−0.0062 (11)
C21	0.0802 (17)	0.0738 (16)	0.0876 (19)	−0.0054 (13)	−0.0323 (15)	0.0069 (14)
C22	0.0842 (18)	0.106 (2)	0.0709 (17)	−0.0246 (17)	−0.0405 (14)	0.0165 (16)
C23	0.0804 (17)	0.104 (2)	0.0514 (13)	−0.0295 (15)	−0.0261 (12)	−0.0049 (13)
C24	0.0553 (12)	0.0755 (15)	0.0457 (11)	−0.0182 (11)	−0.0141 (9)	−0.0107 (10)
C25	0.0486 (12)	0.0704 (14)	0.0597 (13)	−0.0066 (11)	−0.0112 (10)	−0.0215 (11)
C26	0.0644 (13)	0.0514 (12)	0.0739 (14)	0.0118 (10)	−0.0336 (11)	−0.0173 (10)
C27	0.0857 (18)	0.0839 (18)	0.113 (2)	0.0301 (15)	−0.0360 (16)	−0.0465 (16)
C28	0.133 (2)	0.0728 (17)	0.090 (2)	0.0009 (16)	−0.0625 (19)	0.0015 (14)
O2	0.070 (5)	0.111 (9)	0.192 (12)	0.006 (4)	−0.033 (6)	−0.099 (9)
C29	0.081 (5)	0.095 (5)	0.094 (5)	−0.007 (3)	−0.031 (4)	−0.025 (4)
C30	0.175 (13)	0.31 (2)	0.089 (7)	−0.110 (12)	−0.025 (7)	−0.046 (9)
O2A	0.064 (7)	0.082 (5)	0.085 (4)	−0.007 (5)	−0.006 (4)	−0.041 (3)
C29A	0.089 (7)	0.106 (8)	0.109 (9)	−0.013 (5)	−0.018 (6)	−0.038 (7)
C30A	0.041 (5)	0.148 (9)	0.186 (14)	−0.015 (5)	0.006 (6)	−0.071 (8)

### Geometric parameters (Å, º)

**Table:**

F1—C11	1.364 (2)	C17—H17A	0.9300
N1—C1	1.384 (2)	C18—C19	1.500 (3)
N1—C14	1.389 (2)	C18—C25	1.532 (3)
N1—C26	1.470 (2)	C18—H18A	0.9800
N2—C15	1.353 (2)	C19—C20	1.365 (3)
N2—N3	1.356 (2)	C19—C24	1.386 (3)
N2—C18	1.451 (2)	C20—C21	1.391 (3)
N3—C17	1.317 (2)	C20—H20A	0.9300
N4—C25	1.344 (3)	C21—C22	1.370 (4)
N4—C24	1.399 (3)	C21—H21A	0.9300
N4—H1N1	0.85 (2)	C22—C23	1.374 (4)
O1—C25	1.218 (3)	C22—H22A	0.9300
C1—C2	1.395 (3)	C23—C24	1.371 (3)
C1—C6	1.406 (3)	C23—H23A	0.9300
C2—C3	1.367 (3)	C26—C27	1.515 (3)
C2—H2A	0.9300	C26—C28	1.519 (3)
C3—C4	1.389 (3)	C26—H26A	0.9800
C3—H3A	0.9300	C27—H27A	0.9600
C4—C5	1.371 (3)	C27—H27B	0.9600
C4—H4A	0.9300	C27—H27C	0.9600
C5—C6	1.398 (3)	C28—H28A	0.9600
C5—H5A	0.9300	C28—H28B	0.9600
C6—C7	1.436 (2)	C28—H28C	0.9600
C7—C14	1.372 (2)	O2—C29	1.474 (13)
C7—C8	1.472 (2)	O2—H1O2	0.99 (9)
C8—C13	1.389 (3)	C29—C30	1.456 (12)
C8—C9	1.390 (3)	C29—H29A	0.9700
C9—C10	1.380 (3)	C29—H29B	0.9700
C9—H9A	0.9300	C30—H30A	0.9600
C10—C11	1.364 (3)	C30—H30B	0.9600
C10—H10A	0.9300	C30—H30C	0.9600
C11—C12	1.361 (3)	O2A—C29A	1.497 (13)
C12—C13	1.375 (3)	O2A—H2O2	0.76 (10)
C12—H12A	0.9300	C29A—C30A	1.438 (14)
C13—H13A	0.9300	C29A—H29C	0.9700
C14—C15	1.466 (2)	C29A—H29D	0.9700
C15—C16	1.370 (3)	C30A—H30D	0.9600
C16—C17	1.388 (3)	C30A—H30E	0.9600
C16—H16A	0.9300	C30A—H30F	0.9600
			
C1—N1—C14	107.55 (15)	C25—C18—H18A	110.2
C1—N1—C26	127.68 (16)	C20—C19—C24	120.1 (2)
C14—N1—C26	123.80 (16)	C20—C19—C18	131.91 (19)
C15—N2—N3	112.64 (15)	C24—C19—C18	107.96 (18)
C15—N2—C18	129.00 (15)	C19—C20—C21	118.2 (2)
N3—N2—C18	117.92 (15)	C19—C20—H20A	120.9
C17—N3—N2	104.16 (15)	C21—C20—H20A	120.9
C25—N4—C24	111.97 (18)	C22—C21—C20	120.9 (3)
C25—N4—H1N1	122.7 (14)	C22—C21—H21A	119.5
C24—N4—H1N1	122.9 (14)	C20—C21—H21A	119.5
N1—C1—C2	130.23 (19)	C21—C22—C23	121.3 (2)
N1—C1—C6	108.26 (15)	C21—C22—H22A	119.4
C2—C1—C6	121.50 (19)	C23—C22—H22A	119.4
C3—C2—C1	117.7 (2)	C24—C23—C22	117.5 (2)
C3—C2—H2A	121.2	C24—C23—H23A	121.3
C1—C2—H2A	121.2	C22—C23—H23A	121.3
C2—C3—C4	121.7 (2)	C23—C24—C19	122.0 (2)
C2—C3—H3A	119.1	C23—C24—N4	128.3 (2)
C4—C3—H3A	119.1	C19—C24—N4	109.66 (18)
C5—C4—C3	121.0 (2)	O1—C25—N4	127.7 (2)
C5—C4—H4A	119.5	O1—C25—C18	124.8 (2)
C3—C4—H4A	119.5	N4—C25—C18	107.5 (2)
C4—C5—C6	119.0 (2)	N1—C26—C27	112.74 (19)
C4—C5—H5A	120.5	N1—C26—C28	110.06 (18)
C6—C5—H5A	120.5	C27—C26—C28	113.7 (2)
C5—C6—C1	119.09 (17)	N1—C26—H26A	106.6
C5—C6—C7	133.46 (18)	C27—C26—H26A	106.6
C1—C6—C7	107.45 (16)	C28—C26—H26A	106.6
C14—C7—C6	106.08 (15)	C26—C27—H27A	109.5
C14—C7—C8	126.39 (16)	C26—C27—H27B	109.5
C6—C7—C8	127.53 (16)	H27A—C27—H27B	109.5
C13—C8—C9	117.72 (17)	C26—C27—H27C	109.5
C13—C8—C7	121.00 (17)	H27A—C27—H27C	109.5
C9—C8—C7	121.27 (17)	H27B—C27—H27C	109.5
C10—C9—C8	121.3 (2)	C26—C28—H28A	109.5
C10—C9—H9A	119.4	C26—C28—H28B	109.5
C8—C9—H9A	119.4	H28A—C28—H28B	109.5
C11—C10—C9	118.5 (2)	C26—C28—H28C	109.5
C11—C10—H10A	120.8	H28A—C28—H28C	109.5
C9—C10—H10A	120.8	H28B—C28—H28C	109.5
C12—C11—F1	118.5 (2)	C29—O2—H1O2	111 (6)
C12—C11—C10	122.39 (19)	C30—C29—O2	104.6 (11)
F1—C11—C10	119.1 (2)	C30—C29—H29A	110.8
C11—C12—C13	118.7 (2)	O2—C29—H29A	110.8
C11—C12—H12A	120.7	C30—C29—H29B	110.8
C13—C12—H12A	120.7	O2—C29—H29B	110.8
C12—C13—C8	121.40 (19)	H29A—C29—H29B	108.9
C12—C13—H13A	119.3	C29—C30—H30A	109.5
C8—C13—H13A	119.3	C29—C30—H30B	109.5
C7—C14—N1	110.64 (16)	H30A—C30—H30B	109.5
C7—C14—C15	128.66 (16)	C29—C30—H30C	109.5
N1—C14—C15	120.57 (15)	H30A—C30—H30C	109.5
N2—C15—C16	105.47 (16)	H30B—C30—H30C	109.5
N2—C15—C14	121.55 (16)	C29A—O2A—H2O2	99 (10)
C16—C15—C14	132.98 (17)	C30A—C29A—O2A	107.1 (13)
C15—C16—C17	105.79 (18)	C30A—C29A—H29C	110.3
C15—C16—H16A	127.1	O2A—C29A—H29C	110.3
C17—C16—H16A	127.1	C30A—C29A—H29D	110.3
N3—C17—C16	111.93 (18)	O2A—C29A—H29D	110.3
N3—C17—H17A	124.0	H29C—C29A—H29D	108.5
C16—C17—H17A	124.0	C29A—C30A—H30D	109.5
N2—C18—C19	114.71 (15)	C29A—C30A—H30E	109.5
N2—C18—C25	108.29 (16)	H30D—C30A—H30E	109.5
C19—C18—C25	102.81 (16)	C29A—C30A—H30F	109.5
N2—C18—H18A	110.2	H30D—C30A—H30F	109.5
C19—C18—H18A	110.2	H30E—C30A—H30F	109.5
			
C15—N2—N3—C17	−0.9 (2)	N3—N2—C15—C16	1.2 (2)
C18—N2—N3—C17	−174.05 (16)	C18—N2—C15—C16	173.39 (17)
C14—N1—C1—C2	−179.41 (19)	N3—N2—C15—C14	−179.04 (15)
C26—N1—C1—C2	−10.4 (3)	C18—N2—C15—C14	−6.9 (3)
C14—N1—C1—C6	1.1 (2)	C7—C14—C15—N2	−82.5 (2)
C26—N1—C1—C6	170.05 (17)	N1—C14—C15—N2	93.0 (2)
N1—C1—C2—C3	−179.1 (2)	C7—C14—C15—C16	97.1 (3)
C6—C1—C2—C3	0.4 (3)	N1—C14—C15—C16	−87.3 (2)
C1—C2—C3—C4	−0.2 (3)	N2—C15—C16—C17	−1.0 (2)
C2—C3—C4—C5	−0.2 (4)	C14—C15—C16—C17	179.33 (18)
C3—C4—C5—C6	0.4 (3)	N2—N3—C17—C16	0.3 (2)
C4—C5—C6—C1	−0.2 (3)	C15—C16—C17—N3	0.5 (2)
C4—C5—C6—C7	180.0 (2)	C15—N2—C18—C19	129.10 (19)
N1—C1—C6—C5	179.39 (16)	N3—N2—C18—C19	−59.1 (2)
C2—C1—C6—C5	−0.2 (3)	C15—N2—C18—C25	−116.7 (2)
N1—C1—C6—C7	−0.7 (2)	N3—N2—C18—C25	55.1 (2)
C2—C1—C6—C7	179.69 (17)	N2—C18—C19—C20	−61.1 (3)
C5—C6—C7—C14	180.0 (2)	C25—C18—C19—C20	−178.4 (2)
C1—C6—C7—C14	0.11 (19)	N2—C18—C19—C24	117.45 (18)
C5—C6—C7—C8	0.8 (3)	C25—C18—C19—C24	0.1 (2)
C1—C6—C7—C8	−179.11 (17)	C24—C19—C20—C21	−1.3 (3)
C14—C7—C8—C13	−40.8 (3)	C18—C19—C20—C21	177.1 (2)
C6—C7—C8—C13	138.22 (19)	C19—C20—C21—C22	0.8 (4)
C14—C7—C8—C9	140.51 (19)	C20—C21—C22—C23	0.2 (4)
C6—C7—C8—C9	−40.4 (3)	C21—C22—C23—C24	−0.8 (4)
C13—C8—C9—C10	0.0 (3)	C22—C23—C24—C19	0.3 (3)
C7—C8—C9—C10	178.73 (18)	C22—C23—C24—N4	−179.4 (2)
C8—C9—C10—C11	1.6 (3)	C20—C19—C24—C23	0.8 (3)
C9—C10—C11—C12	−1.7 (3)	C18—C19—C24—C23	−177.95 (19)
C9—C10—C11—F1	179.68 (19)	C20—C19—C24—N4	−179.49 (18)
F1—C11—C12—C13	178.76 (18)	C18—C19—C24—N4	1.8 (2)
C10—C11—C12—C13	0.1 (3)	C25—N4—C24—C23	176.4 (2)
C11—C12—C13—C8	1.6 (3)	C25—N4—C24—C19	−3.3 (2)
C9—C8—C13—C12	−1.7 (3)	C24—N4—C25—O1	−175.0 (2)
C7—C8—C13—C12	179.65 (17)	C24—N4—C25—C18	3.3 (2)
C6—C7—C14—N1	0.55 (19)	N2—C18—C25—O1	54.6 (3)
C8—C7—C14—N1	179.78 (16)	C19—C18—C25—O1	176.3 (2)
C6—C7—C14—C15	176.46 (17)	N2—C18—C25—N4	−123.82 (18)
C8—C7—C14—C15	−4.3 (3)	C19—C18—C25—N4	−2.0 (2)
C1—N1—C14—C7	−1.0 (2)	C1—N1—C26—C27	62.8 (3)
C26—N1—C14—C7	−170.54 (16)	C14—N1—C26—C27	−129.9 (2)
C1—N1—C14—C15	−177.30 (15)	C1—N1—C26—C28	−65.4 (3)
C26—N1—C14—C15	13.2 (3)	C14—N1—C26—C28	102.0 (2)

### Hydrogen-bond geometry (Å, º)

**Table:**

*D*—H···*A*	*D*—H	H···*A*	*D*···*A*	*D*—H···*A*
N4—H1*N*1···O2^i^	0.85 (2)	1.92 (3)	2.750 (19)	165 (2)
O2—H1*O*2···O1^ii^	0.98 (9)	1.67 (9)	2.650 (2)	172 (11)

Symmetry codes: (i) −*x*+1, −*y*+1, −*z*+1; (ii) *x*, *y*−1, *z*.
